# Deregulation of the pRb-E2F4 axis alters epidermal homeostasis and favors tumor development

**DOI:** 10.18632/oncotarget.12362

**Published:** 2016-09-30

**Authors:** Clotilde Costa, Mirentxu Santos, Mónica Martínez-Fernández, Corina Lorz, Sara Lázaro, Jesús M. Paramio

**Affiliations:** ^1^ Unidad de Oncología Molecular, CIEMAT (ed70A), 28040 Madrid, Spain; ^2^ Unidad de Oncología Molecular y Celular, Instituto de Investigaciones Biomed, Hospital Universitario 12 de Octubre, 28041 Madrid, Spain; ^3^ Present address: Unidad Mixta Roche-Chus, Hospital Universitario, 15706 Santiago de Compostela, Spain

**Keywords:** epidermis, Rb, E2F4, mouse, cancer

## Abstract

E2F/RB activity is altered in most human tumors. The retinoblastoma family of proteins plays a key role in regulating the progression of the cell cycle from the G1 to S phases. This is achieved through negative regulation of E2F transcription factors, important positive regulators of cell cycle entry. E2F family members are divided into two groups: activators (E2F1-E2F3a) and repressors (E2F3b-E2F8). E2F4 accounts for a large part of the E2F activity and is a main E2F repressor member *in vivo*. Perturbations in the balance from quiescence towards proliferation contribute to increased mitotic gene expression levels frequently observed in cancer. We have previously reported that combined *Rb1*-*Rbl1* or *Rb1*-*E2f1* ablation in epidermis produces important alterations in epidermal proliferation and differentiation, leading to tumor development. However, the possible roles of E2F4 in this context are still to be determined. Here, we show the absence of any discernible phenotype in the skin of mice lacking of *E2f4*. In contrast, the inducible loss of *Rb1* in the epidermis of E2F4-null mice produced multiple skin abnormalities including altered differentiation and proliferation, spontaneous wounds, carcinoma *in situ* development and stem cell perturbations. All these phenotypic alterations are associated with extensive gene expression changes, the induction of c-myc and the Akt activation. Moreover the whole transcriptome analyses in comparison with previous models generated also revealed extensive changes in multiple repressive complexes and in transcription factor activity. These results point to E2F4 as a master regulator in multiple steps of epidermal homeostasis in Rb1 absence.

## INTRODUCTION

In the adult organism, tissue homeostasis and regulation of cell fate decisions, such as whether to divide or differentiate, are key issues in physiological and developmental processes. The unbalance of these events leads to different pathologies including tumor development. In the epidermis there is constant renewal to ensure continued supply of newly differentiated cells, and there is a perfect balance between proliferation and differentiation to guarantee that these orchestrated events work properly [1]. Thus, epidermis is the perfect scenario as a model to study both processes, and to define the role of cell cycle regulators in tumor development.

During epidermal differentiation, cells are characterized by the repression of multiple proliferation-associated genes leading to permanent cell cycle exit and the activation of cell type-specific genes, which confer specific properties to the differentiated cells. Moreover, keratinocytes may respond to cell cycle deregulation and DNA damage by triggering terminal differentiation [2]. Multiple genes involved either in cell cycle exit or in differentiation, are modulated by the Retinoblastoma and E2F family members. Indeed, E2Fs transcription factors play a pivotal role in cell proliferation and differentiation through the binding to retinoblastoma family proteins (pRb, p107 and p130). The E2F family of transcription factors is formed by 8 members (E2F1-8) and is divided into activators (E2F1, E2F2, E2F3a) and repressors (E2F3b-E2F8) [3]. Remarkably, *in vitro* differentiation studies revealed the differential expression and functionally co-operative roles for the retinoblastoma family of proteins in epidermal differentiation [4], whereas E2F1 and E2F4 appeared to exert opposite functions in this system [5].

Using various mouse models, we have previously defined key roles for the Retinoblastoma family in epidermal homeostasis, proliferation, differentiation and tumor development (reviewed in Costa *et al* [6]). We described the existence of multiple compensatory mechanisms through which the absence of one gene is partially compensated by other member. This affects multiple and diverse processes and thus the deletion of two or more related genes produces a panoply of phenotypes, which depend on the specific genes ablated [7–12]. We have observed that deletion of either *Rbl1* or *E2f1* gene in a background of inducible epidermal loss of *Rb1* (Rb^f/f^;K14creER^TM^;p107^−/−^ or Rb^f/f^;K14creER^TM^;E2F1^−/−^ mice, respectively) leads to aggravation of the phenotype caused by *Rb1* loss (observed both in Rb^f/f^;K14cre and in Rb^f/f^;K14creER^TM^ mice) allowing spontaneous tumor development [10, 13]. Of note, this spontaneous tumorigenesis was not observed in mice lacking pRb either in a constitutive or inducible manner [8, 10], whereas skin chemical carcinogenesis in mice lacking *Rb1* in epidermis (Rb^f/f^;K14cre) rendered reduced size and number of tumors, but with more aggressive characteristics compared with control mice [7, 14]. Nonetheless, the location and characteristics of spontaneous carcinomas arising in Rb^f/f^; K14creER^TM^;p107^−/−^and Rb^f/f^;K14creER^TM^;E2F1^−/−^ mice are different. In Rb^f/f^;K14creER^TM^;p107^−/−^ mice, all animals developed differentiated carcinomas almost exclusively located in facial and perioral areas [11]. In contrast, carcinomas developed in Rb^f/f^;K14creER^TM^;E2F1^−/−^ mice are predominantly located in body fur, display follicular origin without expression of interfollicular differentiation markers, have incomplete penetrance and slow progression with no overt signs of malignancy [10]. Accordingly, tumor development in these two models occurs by different molecular mechanisms. In Rb^f/f^; K14creER^TM^;p107^−/−^ mice, the tumors were characterized by activation of Akt/mTOR axis signaling [11], whereas in Rb^f/f^;K14creER^TM^;E2F1^−/−^ mice we observed aberrant β-catenin signaling [10]. We have also observed that the downregulation of *E2F4* gene is a common feature between Rb^f/f^;K14creER^TM^;p107^−/−^ and *Rb1*-deficient keratinocytes, whilst *E2F4* expression is induced in the absence of *E2F1* regardless of *Rb1* status [10, 11]. Importantly, E2F4 bound to pRb, p107 or p130 accounts for the main E2F repressor activity *in vivo* [15, 16], and different E2F4 containing complexes are observed during *in vitro* differentiation of human keratinocytes [5]. In this regard, E2F4 loss suppresses tumorigenesis in pRb mutant mice in part due to the generation of aberrant complexes between p107 and p130 and E2F activator members [15]. In addition, the extensive gene expression rewiring occurring in mice lacking *Rb1* and *Rbl2* in epidermis is partially mediated by altered localization of E2F1 and E2F4 [12]. These observations led us to study the consequences of generalized E2F4 loss in skin. Since these mice did not display any overt phenotype, we also have studied the functional consequences of E2F4 loss in a context of inducible, epidermal-specific, *Rb1* loss. Our present data revealed that E2F4 exerts relevant roles in epidermis in absence of pRb, including tumor suppressive functions.

## RESULTS AND DISCUSSION

To study the possible roles of E2F4 in skin and the epidermal consequences of acute loss of *Rb1* in *E2f4* absence, we generated an inducible mutant mouse model using K14creER^TM^ tamoxifen dependent recombination [10] in a null background for *E2f4* [17], from now on: Rb^f/f^;K14creER^TM^;E2F4^−/−^.

### E2F4 loss aggravates epidermal and skin abnormalities in acute Rb1 mutant mice

**Figure 1:**
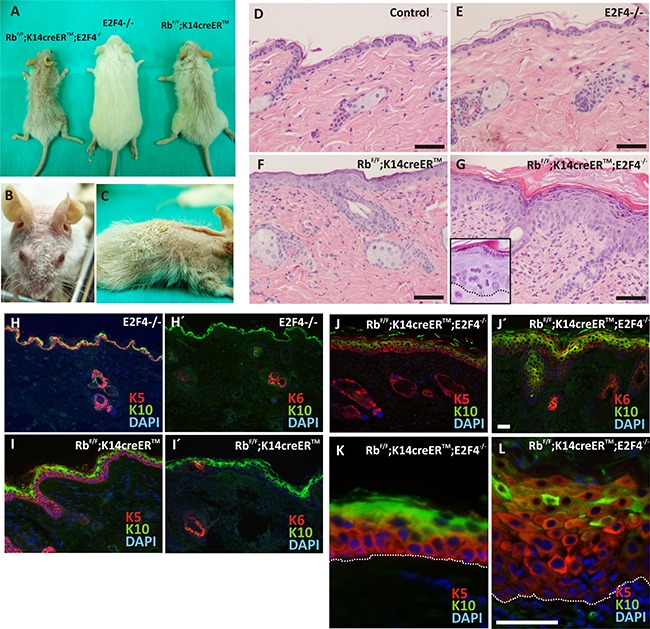
Phenotypic characterization of Rb^F/F^;K14creER^TM^;E2F4^−/−^ mice **A.** Gross appearance of the Rb^F/F^;K14creER^TM^;E2F4^−/−^; E2F4^−/−^ and Rb^F/F^;K14creER^TM^ mice 12 months after topical tamoxifen treatment. **B, C.** Macroscopic aspect of Rb^F/F^;K14creER^TM^;E2F4^−/−^ head and back respectively. **D-G.** H&E stained skin sections of back skin samples of Control (D); E2F4^−/−^ (E); Rb^F/F^;K14creER^TM^ (F) and Rb^F/F^;K14creER^TM^;E2F4^−/−^ (G) mice. **H-L.** Representative double immunofluorescence of K5 or K6 (red) and K10 (green) of the quoted genotypes. Nuclei are stained in blue with DAPI. Bars = 100 μm (H-J Bars =50 μm).

*E2f4* KO mice did not display any overt phenotype in skin (Figure 1A), similarly to E2F1-null mice [10]. This is in contrast with the reported phenotype caused by inducible *Rb1* loss in epidermis, leading to sparse hair, altered epidermal proliferation and differentiation [6, 10]. Strikingly, Rb^f/f^;K14creER^TM^;E2F4^−/−^ mice display a progressive phenotype, which, in the most aggressive cases (6/20 mice), was characterized by decreased growth, reduced or absent hair (predominantly in the head areas), and a hyperkeratotic flaky skin (Figure 1A, B, C). Histology analyses demonstrated that, compared with controls (Rb^f/f^ and untreated Rb^f/f^;K14creER^TM^ mice) (Figure 1D), the E2F4-null mice had no obvious alterations (Figure 1E), whereas, as previously reported [6, 10], the inducible loss of *Rb1* in epidermis produced moderate hyperplasia (Figure 1F). The epidermis of Rb^f/f^; K14creER^TM^;E2F4^−/−^ mice was drastically altered (Figure 1G), showing generalized hyperplasia, hyperkeratosis, dysplasia and suprabasal misoriented mitosis (Inset in Figure 1G).

Regarding epidermal differentiation, we found that, compared to control (Figure 1H, H') and *E2f4*-null mice (not shown), the expression of keratins K5, K10 and K6 is altered in Rb^f/f^;K14creER^TM^ and Rb^f/f^;K14creER^TM^;E2F4^−/−^ mice. In agreement with our previous observations [6, 10], the induction of *Rb1* loss in epidermis caused a mild expansion of K5 expressing cells (Figure 1I) to suprabasal layers, where it is co-expressed with keratin K10, and also the induction of keratin K6 in patches of interfollicular epidermis (Figure 1I'). These alterations are dramatically aggravated in Rb^f/f^;K14creER^TM^;E2F4^−/−^ epidermis, showing an increased number of epidermal layers expressing K5 with reduced K10 expression (Figure 1J–1L), and induction of K6 throughout the entire interfollicular epidermis (Figure 1J'). Structural proteins of stratum corneum (loricrin and filaggrin) have a discontinued pattern that correlated with the reduction of K10 (Supplementary Figure S1A). These data indicate that the loss of *Rb1* in the absence of *E2f4* caused impaired epidermal differentiation, similar to those observed in Rb^f/f^;K14creER^TM^;p107^−/−^ and Rb^f/f^;K14creER^TM^;E2F1^−/−^ mice [6, 10]. However, in sharp contrast with these previously described mouse models and with all the other mice in the present study, including tamoxifen treated Rb^f/f^;K14creER^TM^ (Figure 1K), we also noticed that the expression of K5 and K14 is drastically reduced in the basal layer of Rb^f/f^;K14creER^TM^;E2F4^−/−^ mouse epidermis (Figure 1L, Supplementary Figure S1B,D), showing a predominant intense expression in suprabasal cells. In addition, filaggrin and loricrin expression is absent in these areas (Supplementary Figure 1B-C), whilst a pankeratin staining, using AE1/AE3 antibodies (Supplementary Figure S1E), indicated a general reduction in keratin expression in some areas of the basal layer of the Rb^f/f^;K14creER^TM^;E2F4^−/−^ epidermis (arrows in Supplementary Figure S1E). These findings indicated that the combined loss of *Rb1* and *E2f4* exerts specific epidermal differentiation functions. Similar alterations in epidermal differentiation have been previously observed in various mouse models in which the loss of Rb1 is combined with deficiency in other genes involved in the so-called pRb-pathway, including p107 [8, 11], E2F1 [10] or p21^cdkn1a^ [9], but in most cases the presence of a single functional *Rb1* allele is sufficient to abrogate these differentiation defects, thus indicating that pRb is a major player in this process, probably through the modulation of the transition between proliferative basal layer and differentiating suprabasal epidermal cells [8].

### Rb-E2F4 disruption leads to spontaneous wounds and epidermal fragility

**Figure 2:**
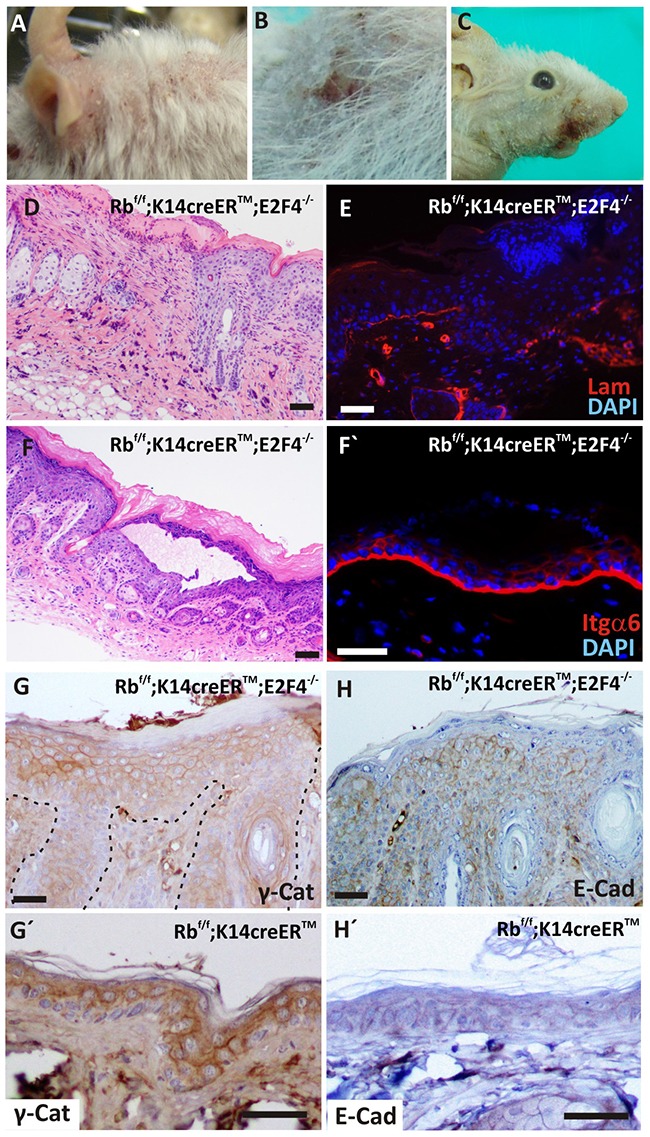
Spontaneous wounds and epidermal fragility in Rb^F/F^; K14creER^TM^; E2F4^−/−^ mice **A-C.** Macroscopic wounds in head (A), back (B) and snout (C) of tamoxifen treated mice. **D.** H&E staining showing epidermal loss in double mutant skin. **E.** Immunofluorescence showing the loss of laminin (red) in epidermal basal lamina. Nuclei are stained in blue (DAPI). **F.** H&E stained section of an epidermal lesion forming blisters. **F'** Immunofluorescence for integrin α6 (red) of Rb^F/F^;K14creER^TM^;E2F4^−/−^ epidermis. Nuclei are stained in blue. **G-H'.** Inmunohistochemistry of hyperplastic Rb^F/F^;K14creER^TM^;E2F4^−/−^ **(G; H)** and Rb^F/F^;K14creER^TM^ (G', H') epidermis for adhesion molecules: γ-catenin **(G, G')** and E-cadherin **(H, H').** Bars= 50 μm (F': Bar = 100 μm)

We observed the appearance of spontaneous wounds in the back and face skin of Rb^f/f^;K14creER^TM^;E2F4^−/−^ mice (Figure 2A-C). The histology of these lesions showed areas of complete epidermal loss (Figure 2D) concomitant with laminin absence (Figure 2E). In addition, we sporadically observed epidermal lesions consisting of material-filled blisters (Figure 2F) affecting exclusively the epidermis, as demonstrated by integrin staining (Figure 2F'), and suggestive of bullous epidermal fragility processes. Similarly, a moderate partial reduction of γ-Catenin and E-cadherin expression was detected in the basal layer of Rb^f/f^;K14creER^TM^;E2F4^−/−^ epidermis (Figure 2G, H, respectively), compared to Rb^f/f^;K14creER^TM^ (Figure 2G', 2H') suggesting a possible loss of adhesion.

Loss of adhesion molecules led to epidermal fragility [18], moreover, K5 and K14 mutations accounts for the majority of Epidermolysis Bullosa Simplex cases [19]. In the basal epidermis of Rb^f/f^;K14creER^TM^;E2F4^−/−^ mice we observed reduction of keratin expression (Figure 1L and Supplementary Figure S1E) and the decrease in adhesion molecules (Figure 2G, 2H), that could account for a possible epidermal fragility and thus spontaneous wound generation.

### Carcinoma *in situ* development in Rb^f/f^; K14creER^TM^;E2F4^−/−^ epidermis

**Figure 3:**
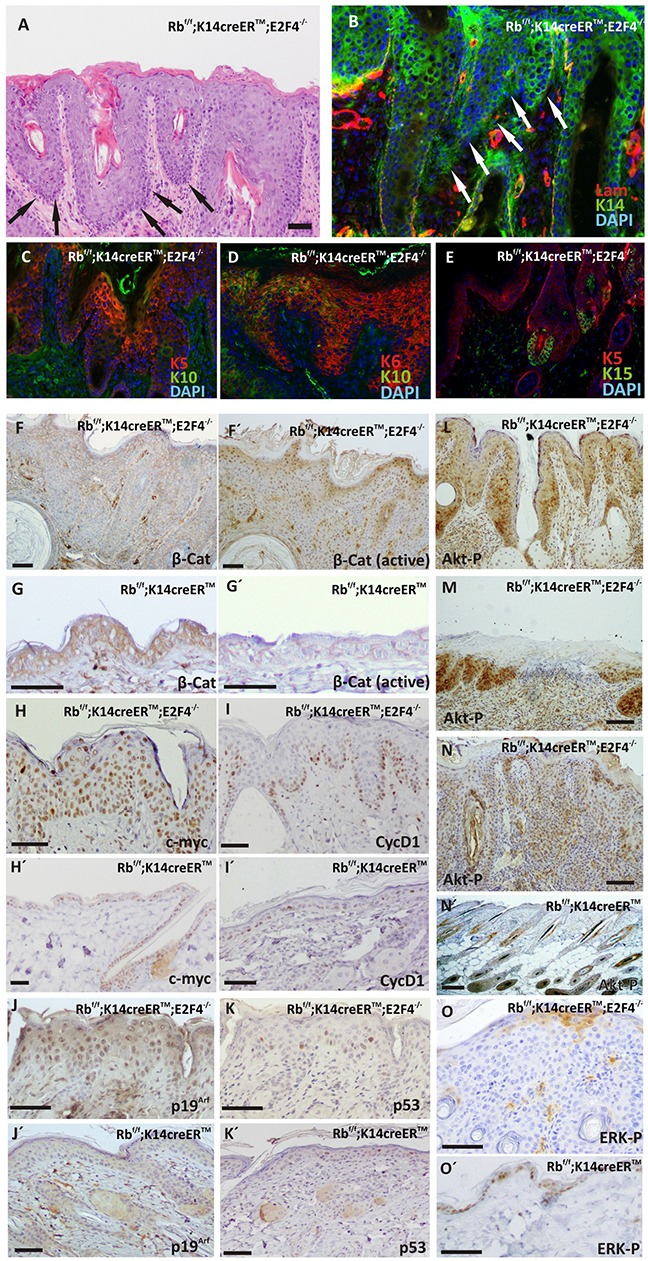
Carcinoma *in situ* development in Rb^F/F^; K14creER^TM^;E2F4^−/−^ mice **A.** H&E stained section of double mutant epidermis showing carcinomas in situ (black arrows). **B-E.** Phenotypic characterization of carcinomas *in situ* present in Rb^F/F^;K14creER^TM^;E2F4^−/−^ epidermis by double immunofluorescence: laminin (red) and K14 (green) (B); K5 (red) and K10 (green) (C); K6 (red) and K10 (green) (D); K5 (red) and K15 (green)(E). Nuclei were stained with DAPI(blue). **F-O.** Representative immunohistochemistry of quoted proteins: total β-catenin expression (F, G); active β-catenin (F′, G′); c-myc **(H, H');** cyclin D1 **(I, I');** p19 (J, J); p53 (K, K); AKT-P^S473^ **L, M, N, N'.** and ERK-P O, O'. in Rb^F/F^;K14creER^TM^;E2F4^−/−^ (F, G, H, I, J, K, L, M. N, O) and Rb^F/F^;K14creER^TM^ **F', G', H', I', J', K', N'.** epidermis. Bars=150 μm.

Histology analyses also revealed areas of massive epidermal hyperplasia (Figure 3A), in which the presence of invasive cells (denoted by arrows in Figure 3A) indicates the existence of possible carcinoma *in situ*, confirmed by the invasion and rupture of basal lamina (Figure 3B). These carcinoma *in situ* were observed in 10 out of 20 mice analyzed and were preferentially localized in the back, snout and eyelid epidermis. They were characterized by the presence of cells expressing K5 (Figure 3C) and K6 (Figure 3D), and extreme reduction of K10 expression (Figure 3C, 3D). We did not detect extensive expression of K15 to interfollicular epidermis, which was limited to the adjacent hair follicles (Figure 3E), in a clear difference with spontaneous hair follicle-derived tumors previously observed in Rb^f/f^;K14creER^TM^;E2F1^−/−^ skin [10]. Although we observed an overall reduction in total βcatenin (Figure 3F), we found a clear increased nuclear localization of active βcatenin (Figure 3F') in areas of carcinoma *in situ* of Rb^f/f^;K14creER^TM^;E2F4^−/−^ mice, compared with the epidermis of all other genotypes, including Rb^f/f^;K14creER^TM^ mice (Figure 3G, 3G'). These tumors were also characterized by extensive c-myc expression (Figure 3H) compared with the scattered basal expression observed in Rb^f/f^;K14creER^TM^ mouse (Figure 3H'). We also observed increased expression of Cyclin D1 in Rb^f/f^;K14creER^TM^;E2F4^−/−^ (Figure 3I) compared to Rb^f/f^;K14creER^TM^ (Figure 3I'), although it was limited to the basal layer (Figure 3I). It is worth mentioning that, in spite of the high penetrance of the tumor lesions, we found no signs of malignant progression to extensive and/or invasive tumors. Such restricted malignancy could be due to p53-mediated tumor suppression as previously reported in other mouse models, including *Rb1* deficient skin under carcinogenesis challenges [7, 20]. We found expression of p19^Arf^ throughout the displastic area (Figure 3J) compared to the sparse staining in Rb^f/f^;K14creER^TM^ epidermis (Figure 3J'). In spite of this observation, the expression of p53 was limited to a few cells of the lesion (Figure 3K), similar to the pattern observed in Rb^f/f^;K14creER^TM^ mice (Figure 3K'). These observations might suggest the existence of functional p53 activities and signaling, and may indicate that the reduced malignancy could be attributed to such functional p53. However, we did not detect any significant signs of apoptosis (analyzed by active caspase 3 expression) in these areas (not shown), thus indicating also a possible impaired p53 function. These aspects will be the subject of future experiments aimed to discern whether the absence of p53 may contribute to increased malignancy in these mice.

The Ras signaling pathway plays major roles in skin carcinogenesis and is also involved in epidermal proliferation and differentiation [21–24]. We thus analyzed whether the appearance of these lesions could be mediated by an aberrant MAPK or Akt signaling. We detected the expression of phosporylated-AKT^S473^ (active Akt) in hyperplasic skin and in the epidermis surrounding spontaneous wounds (Figure 3L, 3M), as well as in carcinoma *in situ* areas (Figure 3N), in sharp contrast with the limited expression of active Akt in specific hair follicle regions observed in Rb^f/f^;K14creER^TM^ (Figure 3N') similar to that observed in wild type mouse skin [25]. On the contrary, the expression of active, phosphorylated ERK observed in control mouse (Figure 3O') was almost completely absent in the hyperplastic areas of Rb^f/f^; K14creER^TM^;E2F4^−/−^ mouse epidermis (Figure 3O).

**Figure 4:**
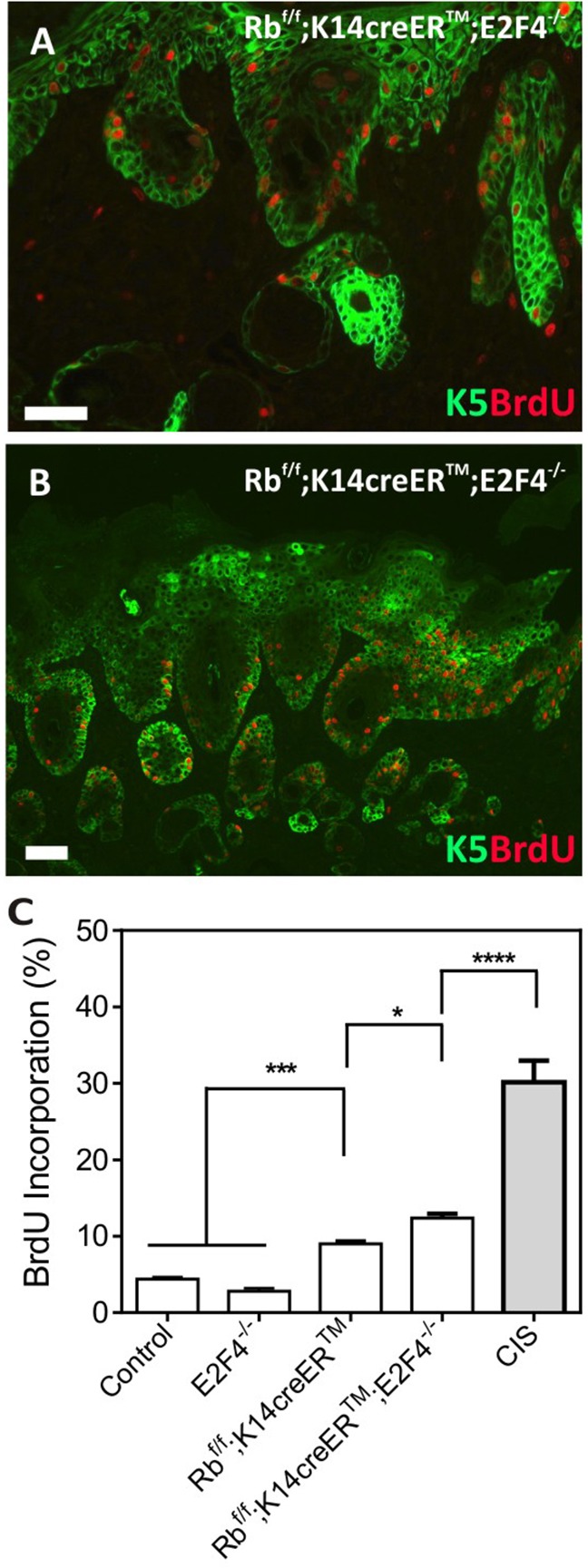
Epidermal Proliferation in Rb^F/F^;K14creER^TM^;E2F4^−/−^ mice **A, B.** Double immunofluorescence showing the expression of K5 (green) and BrdU (red) incorporation in the Rb^F/F^;K14creER^TM^;E2F4^−/−^ epidermis (A) and in carcinoma in situ areas (B). Bars=150 μm **C.** Quantitative analysis of BrdU incorporation in epidermis of the quoted genotypes. Data come from at least three mice per genotype scoring three different sections per mouse; data are shown as mean±s.e. (p values are denoted by asterisks: * p<0.05, *** p<0.005, **** p<0.0001 analyzed by unpaired Mann-Whitney t Tests).

Regarding epidermal proliferation, we found no significant differences in BrdU incorporation between control and E2F4-null mice, whereas, in agreement with our previous data [8, 10, 11], Rb^f/f^;K14creER^TM^ epidermis displayed a significant increase in proliferation (Figure 4C). Such augmented proliferation is further increased in Rb^f/f^;K14creER^TM^;E2F4^−/−^ epidermis (Figure 4A, 4C). Of note, the proliferation is enhanced in the carcinoma *in situ* areas compared with non-lesional epidermis of Rb^f/f^;K14creER^TM^;E2F4^−/−^mice (Figure 4B, 4C).

Collectively the present data demonstrate that the inducible loss of *Rb1* in epidermis of mice lacking *E2f4* is sufficient to cause epidermal fragility, wounds and epidermal carcinomas. The spontaneous tumors were characterized by increased proliferation, nuclear βcatenin accumulation as well as c-myc and AKT-P aberrant expression in damaged Rb^f/f^;K14creER^TM^;E2F4^−/−^ epidermis. These alterations were not observed in mice upon inducible epidermal *Rb1* loss, thus these data indicated that E2F4 and pRb cooperate to maintain epidermal proliferation, integrity and to suppress spontaneous tumor development primarily driven by AKT. This is in accordance with our previous data [26] regarding an increased expression of targets of the βcatenin pathway as c-myc and CycD1 due to Akt/mTOR activation. In addition, the absence of overt malignant progression of the tumors, could be attributable to the reduced ERK activation [27] as well as the activation of p53-dependent processes [7, 28].

### Disruption of Rb/E2F4 axis alters epidermal stem cell homeostasis

Rb^f/f^;K14creER^TM^;E2F4^−/−^ epidermis showed aberrant hair follicles and sebaceous glands (Supplementary Figure S2A-B'). We also observed altered hair cycle in these mice compared to age-matched control or *E2f4^−/−^* mice, as demonstrated by the higher proportion of animals with hairs in anagen (Supplementary Figure S2C). These findings would be in agreement with the aberrant βcatenin nuclear expression observed, as the anagen phase is primarily mediated by active βcatenin [29], and would suggest impaired functionality of the epidermal stem cell compartment in Rb^f/f^;K14creER^TM^;E2F4^−/−^ mice [1, 30, 31], accounting for a possible imbalance of epidermal homeostasis.

**Figure 5:**
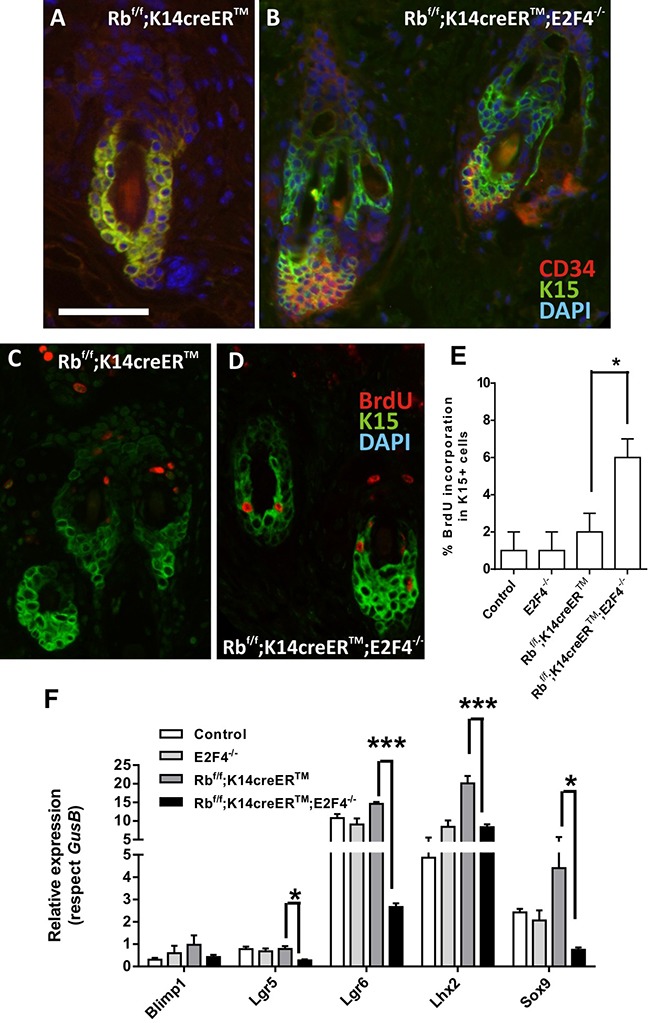
Epidermal stem cell niche is altered in Rb^F/F^;K14creER^TM^;E2F4^−/−^ mice **A, B.** Representative double immunofluorescence images showing the expression of the cell stem markers K15 (green) and CD34 (red) in hair follicles of Rb^F/F^; K14creER^TM^ (A) and Rb^F/F^;K14creER^TM^;E2F4^−/−^ (B) respectively. Nuclei were stained with DAPI (blue). **C-D.** Double immunofluorescence showing BrdU incorporation (red) in the K15 positive cells (green) in Rb^F/F^;K14creER^TM^ (C) and Rb^F/F^;K14creER^TM^;E2F4^−/−^ (D) epidermis. Bars= 150 μm **E.** Quantitative analysis of BrdU incorporation in K15+ cells of the quoted genotypes. Data come from at least five mice per genotype scoring three different sections per mouse and are shown as mean±s.e. **F.** Quantitative analysis of the relative expression of different genes involved in epidermal stem cells homeostasis (*Lgr5*, *Lgr6*, *Blimp1*, *Lhx2* and *Sox9*) in the quoted genotypes (n=6) using qRT-PCR. GusB gene was used as a control for normalization. Samples come from total skin and are shown as mean±s.e.m. (p values in E, F are denoted by asterisks: * p<0.05, *** p<0.005 analyzed by unpaired Mann-Whitney t Tests).

To study possible alterations in bulge epidermal stem cells, we first analyzed the expression of stem cell markers K15 and CD34 by double immunofluorescence. No significant differences were observed among control, E2F4^−/−^ and Rb^f/f^;K14creER^TM^ mouse skin sections (Figure 5A and data not shown), in agreement with our previous data [32, 33]. In contrast, in Rb^f/f^; K14creER^TM^;E2F4^−/−^ hair follicles, the population of K15-expressing cells is partially expanded, resulting in a group of K15+CD34- cells (Figure 5B). In addition, we observed increased proliferation in K15-positive cells in Rb^f/f^;K14creER^TM^;E2F4^−/−^ compared with control, E2F4^−/−^ and Rb^f/f^;K14creER^TM^ mice by BrdU incorporation assay (Figure 5C, 5D, 5E).

We next studied the expression of various genes considered stem cell markers, which also affect the functionality of this cell population. The expression of the *Lhx2*, *Lgr5*, *Lgr6* and *Sox9* were downregulated in Rb^f/f^; K14creER^TM^;E2F4^−/−^ epidermis (Figure 5E). *Lhx2* acts as a transcription factor necessary to specify and maintain hair follicle stem cells, and mediates various signals required to drive activated stem cells to terminally differentiate [34], whereas *Sox9* is implicated in hair differentiation [35]. The observed decreased expression of these genes may account for the described phenotype of Rb^f/f^;K14creER^TM^;E2F4^−/−^ mice regarding hair loss and aberrant anagen entry. *Lgr6* is a bona fide epidermal stem cell marker [36] particularly involved in wound repair as well as in the formation of new hair follicles [37]. Importantly, the Lgr6-positive stem cell pool is Wnt-independent and can renew sebaceous cells and epidermis throughout life [36]. *Lgr5* also contributes to maintain all cell lineages of the hair follicle over long periods of time [38] and Lgr5-positive cells are possible cancer initiating cells in HPV-mediated skin carcinogenesis accounting for increased malignancy [39].

**Figure 6:**
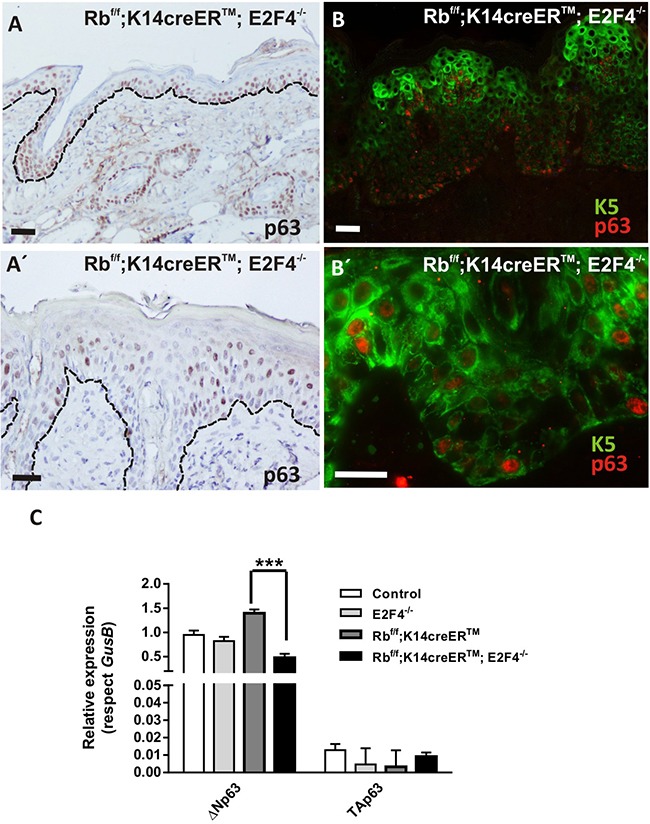
p63 shows an aberrant expression pattern in Rb^f/f^;K14creER^TM^;E2F4^−/−^ mouse epidermis **A, A'.** Representative immunohistochemistry of p63 expression. **B, B'.** Double immunofluorescence showing K5 (green) and p63 (red) in Rb^f/f^;K14creER^TM^;E2F4^−/−^ epidermis. Bars= 50 μm. **C.** Quantitative analysis of the relative expression of p63 (ΔNp63 and TAp63) in the quoted genotypes by qPCR (n=6). *GusB* gene was used as a control for normalization. Samples come from total skin and are shown as mean±s.e.m. (p values are denoted by asterisks: *** p<0.005, analyzed by unpaired Mann-Whitney t Tests).

In line with the altered stem cell population is the aberrant expression and localization of p63. p63 regulates proliferative potential of the basal and stem cell compartment in various epithelial adult tissues, being a major regulator of epidermal homeostasis, and having an important role controlling the expression of genes involved in epidermal cell adhesion [40–42]. Given the observed alterations in adhesion molecules and putative defects in epidermal stem cells of Rb^f/f^; K14creER^TM^;E2F4^−/−^ mice, we sought to study possible defects in p63 expression. Immunohistochemistry studies revealed that, the epidermis of Rb^f/f^;K14creER^TM^;E2F4^−/−^ mice compared with control, E2F4^−/−^ and Rb^f/f^;K14creER^TM^ epidermis (Figure 6A and data not shown) displayed p63 expression expanded to suprabasal layers but partially lost in epidermal disorganized areas (Figure 6A'). In agreement with immunohistochemistry, the study of K5 and p63 expression by double immunofluorescence revealed areas in which p63 is expressed in basal cells with reduced or no expression of K5 (Figure 6B), and the presence of cells expressing K5 that lack p63 in the basal layer of the epidermis (Figure 6B'). Moreover, specific qRT-PCR experiments demonstrated an overall reduction of ΔNp63 isoform expression in Rb^f/f^;K14creER^TM^;E2F4^−/−^skin (Figure 6C). The existence of these populations (K5+p63+; K5+p63- and K5-p63+) might represent different stages in the progression of the phenotype from K5+p63+ towards the aberrant K5+p63- and K5-p63+ epidermal cells. The reduced expression of K5 or p63 may affect epidermal resilience or expression of adhesion molecules [40–42] favoring epidermal fragility. Moreover, since uncontrolled p63 expression could result in the induction of metaplasia or premalignant disease [43, 44], these alterations could also favor spontaneous tumor development.

Collectively, these findings indicate that the inducible loss of *Rb1* in the epidermis of *E2f4^−/−^* mice exerts disturbances in epidermal stem cell population affecting homeostasis. At present, we cannot discard other possible alterations in epidermal stem cell functionality, as the reduced survival and the different penetrance of the epidermal phenotype, preclude the realization of functional assays, such as experimental wound healing and adult keratinocyte clonogenicity determinations. The possible role of Rb-dependent axis on epidermal stem cell homeostasis has been previously suggested. We previously reported that the functional quiescence characteristic of this cell population, in absence of external stimuli, is partially dependent on a functional Rb pathway [32]. In addition, gene profiling revealed that these cells compared with other basal cells in mouse epidermis display reduced expression of various activator E2Fs, such as *E2f1* and *E2f3* [32]. Moreover, the absence of *Rb1* in epidermis is sufficient to alter the quiescent status of epidermal stem cells, although without altering their functionality [32]. The simultaneous absence of *Rb1* and *E2f1* caused the development of spontaneous tumors of hair follicle origin, thus pointing to altered stem cell homeostasis, also supported by the altered expression of various stem cell markers [10]. The absence of histological alterations in the epidermis of *E2f4*-null mice would indicate the lack of relevance of this protein in the mouse epidermal stem cell population. However, this could be also due to functional compensation by other E2F repressor members (see below) or to the presence of other repressor complexes composed by any Rb relative with other E2F members [15, 16]. The presence of such functional complexes in the epidermal stem cell populations of the different mouse models, similar to those previously observed in differentiating human keratinocytes [5], would deserve future investigation. Nonetheless, our present data indicated that the absence of *Rb1* and *E2F4* caused altered functionality of the stem cell population, thus pointing to a supportive role of the Rb/E2F4 axis in stem cell homeostasis in epidermis. Remarkably, such altered homeostasis may also provide molecular basis for the phenotypic alterations observed in Rb^f/f^; K14creER^TM^;E2F4^−/−^ mouse epidermis. Given that the Rb pathway may also be involved in the regulation of p63 family [45–47], the possibility that alterations in epidermal stem cells could also lead to the aberrant p63 expression observed would deserve future research.

### Genome-wide transcriptome analysis of Rb^f/f^; K14creER^TM^;E2F4^−/−^ skin

**Figure 7:**
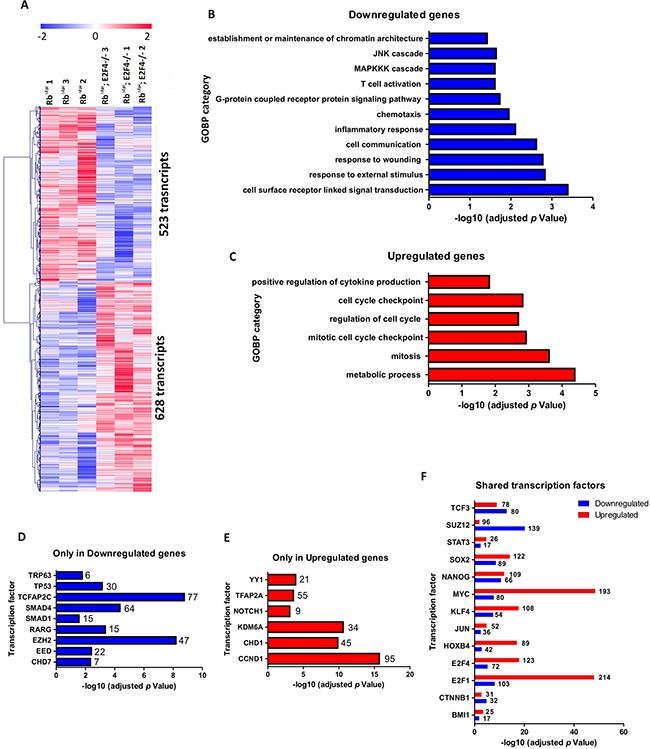
Genome-wide transcriptome comparison between Rb^f/f^;K14creER^TM^;E2F4^−/−^ and Rb^f/f^;K14creER^TM^ skin **A.** Heatmap of the microarray analyses showing 523 transcripts upregulated and 628 transcripts downregulated in Rb^f/f^;K14creER^TM^;E2F4^−/−^ compared to Rb^f/f^;K14creER^TM^ skin. **B, C.** Enrichment analyses of Gene Ontology of the downregulated (B) and upregulated (C) genes. **D, E.** ChEA analyses showing the transcription factors exclusively regulating the downregulated (D) and upregulated genes (E). **F.** ChEA analyses showing the transcription factors involved in the regulation of both upregulated and downregulated genes.

We have previously reported deep changes in skin transcriptome as a consequence of epidermal ablation of *Rb1* alone or in combination with multiple related genes such as *Rbl1*, *Rbl2*, *E2f1*, *p53* and *Cdkn1a* [9–12, 28]. Consequently, we analyzed the gene expression changes in Rb^f/f^;K14creER^TM^;E2F4^−/−^ skin. The comparison between Rb^f/f^;K14creER^TM^ and Rb^f/f^;K14creER^TM^;E2F4^−/−^ skin samples revealed 523 transcripts upregulated and 628 transcripts downregulated in Rb^f/f^; K14creER^TM^;E2F4^−/−^ (Figure 7A and Supplementary Table S1). The downregulated genes were primarily involved in signal transduction and stress response, including wound and terminal differentiation, as well as different processes relative to inflammation (Figure 7B) (*Ctgf, Ccl22, Ccr3, Ccr6, Ccr7, Cx3cl1, Cxcl12, Elf3, Erbb3, F2rl2, Gpr68, Klrg1, Ly75, Nfe2l1, Opn1sw, Pglyrp1, Plekhb1, Sigirr, Xcl1, Xcr1*), which may account for the observed development of epidermal fragility and wounds noted. On the other hand, the upregulated genes (Figure 7C) were primarily involved in metabolic processes and, interestingly, in various aspects of mitosis and mitotic regulation (*Map3k9, Gng3, Bccip, Gna15, Btrc, Hif1a, Traf6, Angptl3, Plk1, Hspd1, Aph1a, Dbnl, Mad2l1, Pxk, Eng, Ccnk, Agt, Psmd12, Pdgfb, Psma5, Blm, Psmb6, Akt1, Psmd2, Psmd6, Plcb2, Bub1b, Cks1b, Map3k2, Ppap2a, Gps1, Cenpe, Cks2*). The analysis of different transcription factors in the modulation of the deregulated genes using the ChEA web-tool [48, 49], revealed that the downregulated genes (Figure 7D) showed a significant and exclusive regulation by various polycomb elements (Ezh2, Eed), Smads, Trp63 and p53. Reinforcing the possible involvement of polycomb repression, the upregulated genes showed, besides others, regulation by Kdm6A (Figure 7E), whose demethylase activity opposes to Polycomb histone methylation. Interestingly, we also observed opposed involvement of Ap2c and Ap2a in gene downregulation and upregulation, respectively. These two transcription factors govern, among other processes, the ordered expression of genes during epidermal differentiation through processes affecting C/EBPs and also E2F family members, and may also influence p63 expression [47]. The ChEA study also provided information about various transcription factors involved in the regulation of both, upregulated and downregulated genes (Figure 7F). Remarkably, E2F4 was predominantly found in the upregulated genes, in agreement with its primary role as gene repression element, and thus its absence leads to increased gene expression.

Interestingly, among the transcription factors regulating genes with increased expression in Rb^f/f^; K14creER^TM^;E2F4^−/−^ compared with Rb^f/f^;K14creER^TM^ skin, we found a predominant involvement of c-myc (Figure 7F) (in agreement with the immunohistochemistry data (Figure 3H)) and E2F1 (Figure 7F). This possible involvement of E2F1, as well as the epidermal phenotypic similarities between Rb^f/f^;K14creER^TM^;E2F4^−/−^ and Rb^f/f^; K14creER^TM^;E2F1^−/−^ mice (aggravated phenotype respect to Rb^f/f^;K14creER^TM^ epidermis, spontaneous wounds and tumors) prompted us to analyze possible differences in whole transcriptome changes between these two mouse models.

**Figure 8:**
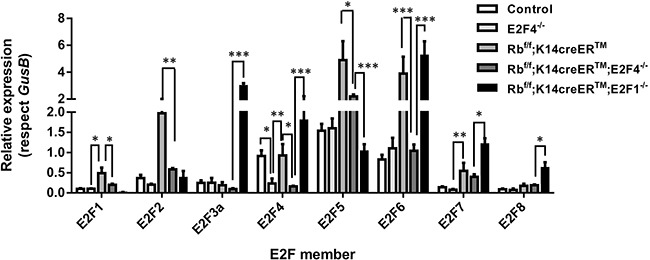
Expression of the E2F transcription factor family Quantitative analysis of the relative expression of E2F family genes (*E2f1*-*E2f8*) in quoted genotypes by qRT-PCR (n=6). *GusB* gene was used as a control for normalization. Samples come from total skin and are shown as mean±s.e.m. (p values are denoted by asterisks: * p<0.05, ** p<0.01, *** p<0.005 analyzed by unpaired Mann-Whitney t Tests).

First, we analyzed the expression of different E2F transcription factors among Rb^f/f^, E2F4^−/−^, Rb^f/f^; K14creER^TM^, Rb^f/f^;K14creER^TM^;E2F4^−/−^ and Rb^f/f^; K14creER^TM^;E2F1^−/−^ mouse skin by RT-qPCR. This analysis revealed that *E2f1* and *E2f2* were significantly and exclusively induced in Rb-deficient skin (Figure 8), similarly, *E2f3a* and *E2f8* were found exclusively induced in Rb^f/f^;K14creER^TM^;E2F1^−/−^ mouse skin. *E2f4* was downregulated, as expected, in *E2f4*-deficient mice (E2F4^−/−^ and Rb^f/f^;K14creER^TM^;E2F4^−/−^). The major changes between both genotypes were observed in *E2f5*, *E2f6*, *E2f7* and *E2f8* repressor factors (Figure 8). As the conventional role of pRb/E2F4 in cell cycle regulation is to act repressing gene expression, such increased expression in repressor E2Fs, together with the observed involvement of various Polycomb members in the repressed genes, would provide a possible explanation of how the absence of this classical repressor complex (Rb-E2F4) can induce gene repression. Also in this regard, a functional interaction between repressor E2F and polycomb members mediating gene repression has been previously reported [50, 51].

**Figure 9:**
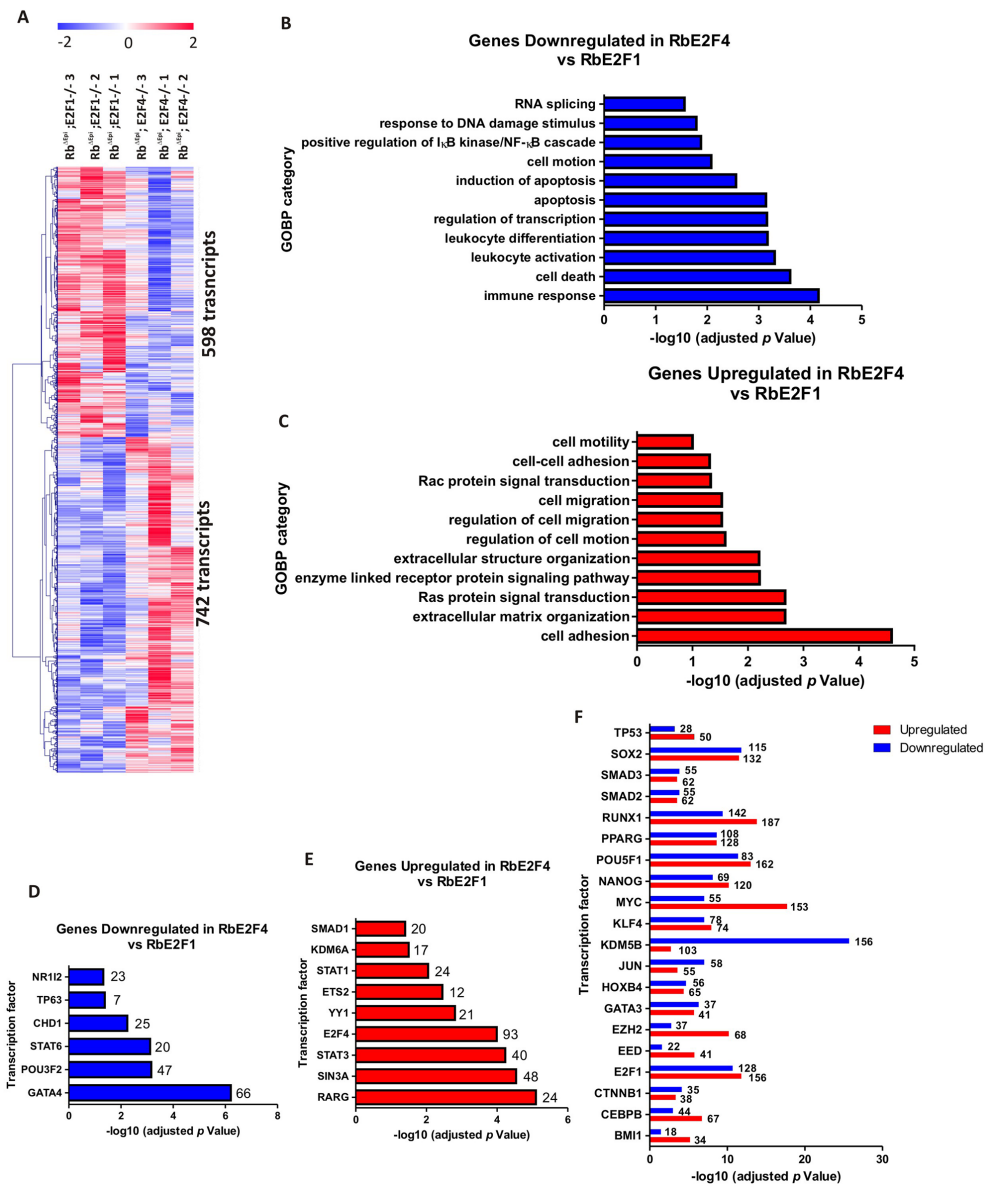
Genome-wide transcriptome comparison between Rb^f/f^;K14creER^TM^;E2F1^−/−^ and Rb^f/f^;K14creER^TM^;E2F4^−/−^ skin **A.** Heatmap of the microarray analyses showed 742 transcripts upregulated and 598 transcripts downregulated in Rb^f/f^; K14creER^TM^;E2F4^−/−^ compared to Rb^f/f^;K14creER^TM^;E2F1^−/^. **B, C.** Enrichment analyses of Gene Ontology of the downregulated (B) and upregulated (C) genes. **D, E.** ChEA analyses showing the transcription factors exclusively regulating the downregulated (D) and upregulated genes (E). **F.** ChEA analyses showing the transcription factors involved in the regulation of both upregulated and downregulated.

The whole transcriptome analysis between Rb^f/f^; K14creER^TM^;E2F4^−/−^and Rb^f/f^;K14creER^TM^;E2F1^−/−^ skin showed the upregulation of 742 and the downregulation of 598 transcripts (Figure 9A and Supplementary Table S2). Downregulated genes were predominantly found to be involved in processes relative to immune response and apoptosis (Figure 9B), whereas upregulated genes were involved in cell adhesion and motility processes, including various signal transduction pathways related to these processes (Figure 9C). The ChEA analysis of transcription factors involved in downregulated (Figure 9D), upregulated (Figure 9E) or both (Figure 9F) revealed a predominant role of c-myc in the gene activation in Rb^f/f^; K14creER^TM^;E2F4^−/−^ skin, and, again, a primarily loss of gene repression mediated by various polycomb members, as well as *E2f4* and Sin3A in Rb^f/f^;K14creER^TM^;E2F4^−/−^ mouse skin, and the possible loss of induction of p63-dependent genes. Of note, we have previously observed that a complex between E2F4 and Sin3A together with p130, HDACs and p27, negatively modulated a large number of genes involved in tumorigenesis [52].

Collectively, our transcriptome analysis revealed that the absence of pRb and E2F4 in epidermis produces severe changes in gene expression with strong differences with those promoted by the absence of pRb or the simultaneous absence of pRb and E2F1. Given the primary role of Rb/E2F4 complexes as a repressive complex, the observed downregulation of genes is particularly interesting. The ChEA analyses indicate that compared to Rb^f/f^;K14creER^TM^, there is a potential involvement of polycomb members in Rb^f/f^;K14creER^TM^;E2F4^−/−^ skin. The relationship between various Rb pathway elements and Polycomb members has been extensively documented. Moreover, the absence of all Rb members is sufficient to induce Ezh2 expression through E2F3, causing gene repression and promoting tumorigenesis in mouse bladder [53]. However, the possible role of E2F4 in this context is presently unknown.

In summary, our data show that acute Rb loss in absence of E2F4 leads to a phenotype characterized by altered terminal differentiation, augmented proliferation, dysplasia, carcinoma *in situ* development, epidermal fragility, wounds, loss of hair and aberrant anagen or sebaceous gland development. Some of these features were never seen before in any of the Rb deficient models previously studied [6, 10, 11], suggesting that Rb/E2F4 axis has a more important role than expected in epidermis homeostasis.

## MATERIALS AND METHODS

### Animals and treatments

Mice were held in our animal facility given food and water ad libitum, in accordance with Centro de Investigaciones Energéticas, Medioambientales y Tecnológicas (CIEMAT) guidelines. Rb^f/f^;K14creER^TM^ mice have been previously described [10]; E2F4^−/−^ mice were obtained from NCI Mouse Repository (B6;129S-E2f4^tm1Lees^; strain code:01XK7). They were backcrossed for at least 10 generations to the inbred strain FVB/N genetic background. E2F4 null mice survival was compromised due to opportunistic infections which were overcome providing antibiotic (Septrin; Celltech, UK) in the drinking water to pregnant female and post-natal mice lifelong. They were smaller at birth than their heterozygous littermates, however, after weaning they were indistinguishable from them. Importantly, 95% of E2F4^−/−^ mice were sterile, thus, crossbreading was done using heterozygous mice [17]. Tamoxifen treatment (Sigma-Aldrich) was topically administered as previously described [10] for 5 consecutive days.

### Immunohistochemical methods

Immunohistochemical or immunofluorescence assays were performed as previously described [10, 11]. Antibodies used were: anti K5 and anti K6(Covance, Princeton, NJ, USA), anti K10 (Dako), anti K15 (NeoMarkers, Fremont, CA, USA), anti CD34 (eBiosciences), anti K17 (kindly provided by Dr. P. Coulombe), anti p63 (Sta Cruz Biotechnology, Santa Cruz, CA, USA), anti-laminin (Sigma), anti βcatenin (Invitrogen, Carlsbad, CA, USA), anti active βcatenin (Millipore, Billerica, MA, USA), anti p53 (Novocastra, Newcastle, UK), anti CyclinD1 (Neomarkers), anti integrinα6 (BD Pharmingen, Franklin Lakes, NJ, USA), anti γ-catenin (BD Transduction Lab), anti E-cadherin, anti c-myc-P^T58/S42^, anti-P-ERK (Cell Signaling, Danvers, MA, USA), anti-AKT-P^S473^, anti-pan-keratin (AE1/AE3) and anti-p19^arf^ (AbCam, Cambridge, UK). Fluorochrome or Biotin-conjugated secondary antibodies were purchased from Jackson ImmunoResearch (West Grove, PA, USA). For immunohistochemistry, signal was amplified using avidin-peroxidase (ABC elite kit Vector, Vector labs., Burlingame, CA, USA) and peroxidase was visualized using diaminobenzidine as a substrate (DAB kit Vector, Vector labs). For proliferation assay, mice were intraperitoneally injected with bromodeoxyuridine (BrdUrd; 0.1 mg/g weight in 0.9% NaCl; Roche, Basel, Switzerland) 1 hour before sacrifice. BrdU incorporation was monitored by double immunofluorescence in ethanol-fixed or in formalin-fixed sections using an anti-BrdU (Roche) and anti K5 antibodies as described [54].

### qRT-PCR

For the qPCR analyses, total RNA was isolated from mice skins using RNeasy Mini Kit (Qiagen) according to the manufacture's recomendations. Genomic DNA was eliminated from the samples by a DNase treatment (Rnase-Free Dnase Set Qiagen). RNA from each sample (800 ng) was reverse transcribed in a final volume of 40 μl using the Omniscript RT Kit (Qiagen) and an oligo (dT)_18_ primer. Real time PCR was performed using SYBR green master mix as previously described [10]. The sequences of the specific oligonucleotides used are as follows (5' to 3'): *muE2F1f*: TGCCAAGAAGTCCAAGAATCA; *muE2F1r*:CTTCAAGCCGCTTACCAATC; *muE2F2f*:TGTGAGCTTGTTCCCACGCTA; *muE2F3f*: CAAGGACC CTCCAGCAGAG; *muE2F3r*: AGTTCCAGCCTTCGCT TTG; *muE2F4f*: GAACTGGACCAGCACAAGGT; *muE2F4r*: CATGAGTCACGTAGGCCAAGC; *muE2F5f*: GCGTCCTGGATCTCAAAGC; *muE2F5r*: GATATCATA ACCACGCCTAGTCCAAGACAA; *muE2F6r*: CCAAC AATCCAGGTTCCATCA; *muE2F7f*: TGTTACGTGAG ACATCCGGTA; *muE2F7r*: GGATGCTCTTGGGAGTCG; *muE2F8f:*GGCATTCGAACATGTGCTTCG; *muE2F8r*: GCTCATCACCGCTAAGGACTT;*muLGR5f*: CTTCACTCGG TGCAGTGCT; *muLGR5r*: CAGCCAGCTACCAAATAG GTG; *muLGR6f*: AGCTTCAGCCGGGTCTCT; *muLGR6r*: AGAGGTGGTTCCCTGAGAGC; *muSOX9f*: CAGCAAG ACTCTGGGCAAG; *muSOX9r*:TCCACGAAGGGTCTC TTCTC; *muBLIMPf*: TGCGGAGAGGCTCCACTA; *muBLIMPr*: TGGGTTGCTTTCCGTTTG; *muLHX2f*: CAG CTTGCGCAAAAGACC*muLHX2r*: TAAAAGGTTGCG CCTGAACT; *muGUSBf*: GAGGATCAACAGTGCCCATT; *muGUSBr*:CAGCCTCAAAGGGGAGGT.

Detection of fluorescence was carried out at the end of each amplification step. Moreover, melting curves were performed to verify specificity of the target and absence of primer dimerization after each amplification. Reaction efficiency was calculated for each primer combination and GUS B gene was used as reference gene.

### Transcriptome analyses

Mouse skin tissue was preserved in RNAlater (Ambion) and disrupted and homogenized using Mixer Mill MM301 (Retsch). Total RNA was extracted and purified from 30 mg of skin using RNeasy Fibrous Tissue Mini kit (Qiagen) following the manufacturers' recommendations. The integrity of the RNA populations was tested in the Bioanalyzer (Agilent). Genome-wide transcriptome experiments were performed using the Affymetrix Mouse GE MOE430 2.0 array or Mo Gene-1_0-st-v1 (Affymetrix, Santa Clara, CA, USA) at the Genomics Facility of the Cancer Research Center (Salamanca, Spain). Datasets have been deposited in GEO (GSE84206/GSE38048) [10]. Analyses were performed essentially as described elsewhere [10]. Briefly, after normalization of the results with RMA Express software, Zscores in log2 scale were calculated for heatmap visualization in each dataset. Data from the different microarray platforms were compared using the “collapse to gene symbol” function in GSEA, obtaining a total of 13435 transcripts. Unsupervised hierarchical clustering was done with Pearson distance metrics and complete linkage method using the open source software Multi experiment viewer (http://www.tm4.org/mev.html). Discrimination between groups were done using SAM using a cutoff of p<0.05 based on 100 randomly permutations and FDR<10%. Enrichment analysis of Gene Ontology terms was done upon uploading selected probe sets identifiers into DAVID Functional Annotation web tool [55]. ChIP Enrichment Analyses were performed using the Chip enrichment analysis web tool (http://amp.pharm.mssm.edu/Enrichr/) without filtering, and transcription factors providing a p-value p< 0.001 were manually curated.

### Statistical analysis

Comparisons were performed using the Wilcoxon–Mann–Whitney test (for two groups) and Student's t Test for paired samples showing normal distribution. SPSS 17.0 and Graph prism 6.0 software were used.

## SUPPLEMENTARY FIGURES AND TABLES






