# Correction: Deregulation of the pRb-E2F4 axis alters epidermal homeostasis and favors tumor development

**DOI:** 10.18632/oncotarget.19511

**Published:** 2017-07-24

**Authors:** Clotilde Costa, Mirentxu Santos, Mónica Martínez-Fernández, Corina Lorz, Sara Lázaro, Jesús M. Paramio

**Present**: The grant support section is incomplete.

**Correct:** The complete grant support information appears below.

Original article: Oncotarget. 2016; 7:75712-75728. https://doi.org/10.18632/oncotarget.12362

**FUNDING**

This work was supported partially by funds from Fondo Europeo de Desarrollo Regional (FEDER) and by grants from the Ministry of Economy and Competitiveness of Spain (ISCIII grants PI12/01959 and PI15/00993 to MS and MINECO grants SAF2012-34378 and SAF2015-66015-R) to JMP. Comunidad Autónoma de Madrid grant S2010/BMD- 2470 (Oncocycle Program), MSyC grant ISCIII-RETIC RD12/0036/0009 and PIE 15/00076 to JMP. MM-F was supported by Juan de la Cierva (JCI-2010-06167).

