# Supplementary material for: Deregulation of the pRb-E2F4 axis alters epidermal homeostasis and favors tumor development
Source: Oncotarget. 2016 Sep 30;7(46):75712–28. doi: 10.18632/oncotarget.12362 (PMC5342772; doi:10.18632/oncotarget.12362)
Supplement: Supplementary file 1 [file oncotarget-07-75712-s001.pdf]

Deregulation of the pRb-E2F4 axis alters epidermal homeostasis and favors tumor development

SUPPLEMENTARY FIGURES AND TABLES

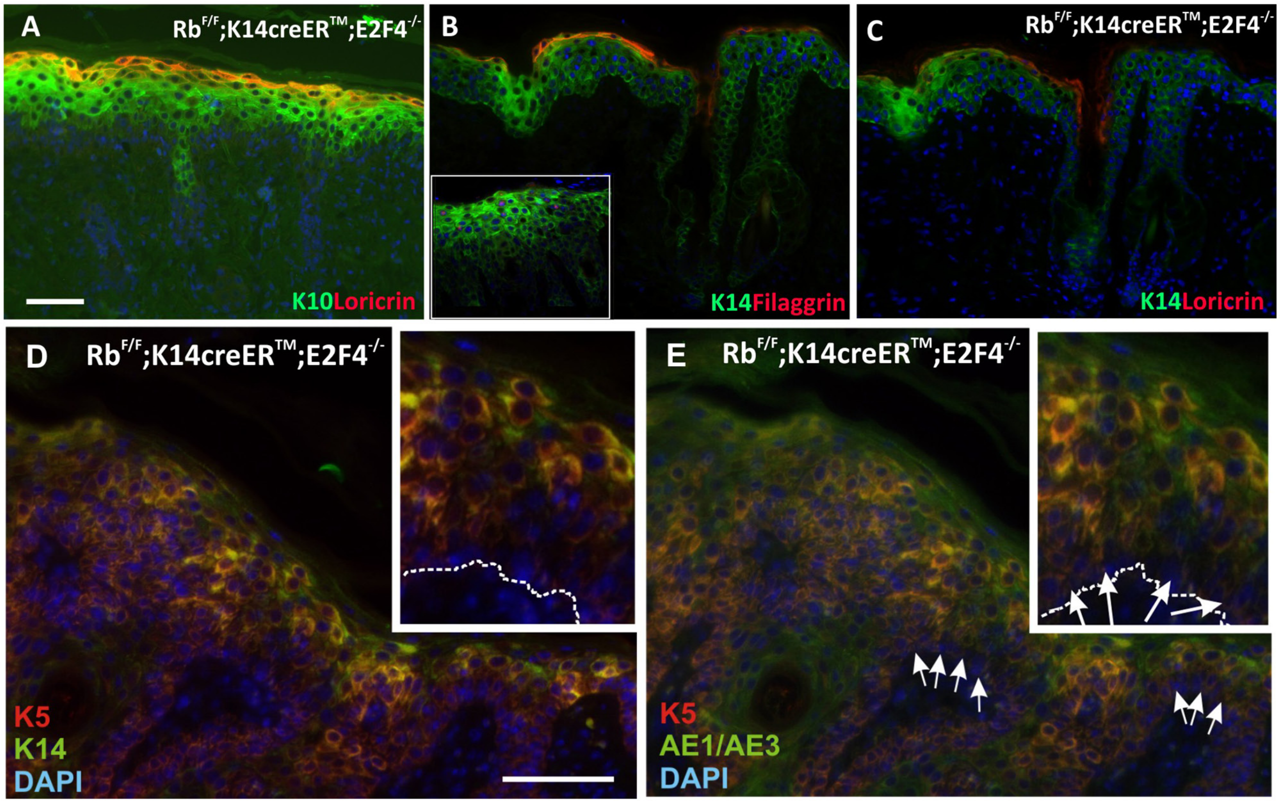

Supplementary Figure S1: Rb$^{F/F}$;K14creER^{TM};E2F4$^{-/-}$ epidermis presents aberrant epidermal differentiation. A-E. Representative immunofluorescence of Rb$^{f/f}$;K14creER^{TM};E2F4$^{-/-}$ epidermis for K10 (green) and loricrin(red) (A); K14 (green) and filaggrin (red) (B); K14 (green) and loricrin (red) (C). stratum corneum proteins present a patched pattern opposite to K14 expression. D) K5 (red) and K14 (green). E) K5 (red) and AE1/AE3 (green). Nuclei were stained with DAPI. Bars= 50 µm.

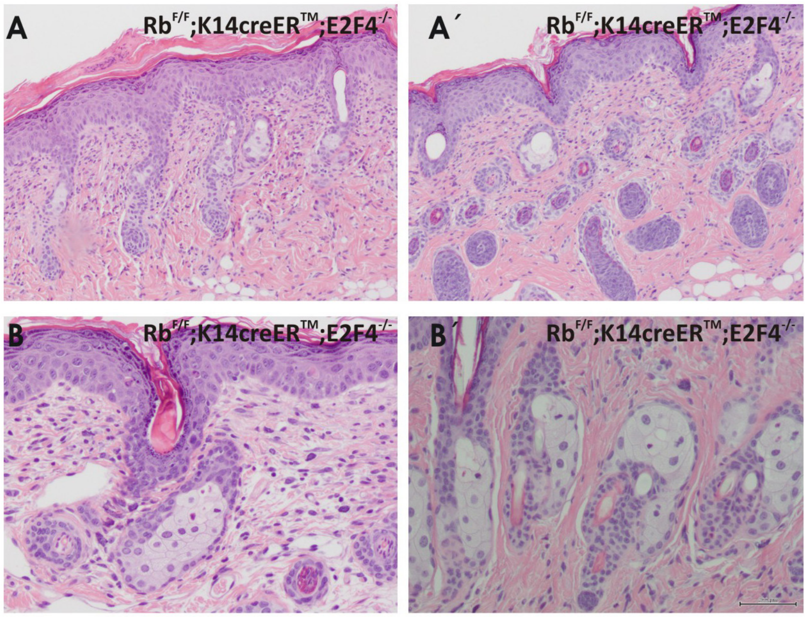

C

	E2F4$^{-/-}$	Rb$^{f/f}$;K14creER^{TM};E2F4$^{-/-}$
**Anagen**	3	10
**Telogen**	9	0

Supplementary Figure S2: Aberrant hair follicles and sebaceous glands in Rb$^{F/F}$;K14creER^{TM};E2F4$^{-/-}$ epidermis. Representative H&E sections of Rb$^{f/f}$;K14creER^{TM};E2F4$^{-/-}$ epidermis showing aberrant anagens **A, A'.** and sebaceous glands **B, B'. C.** Number of mice with aberrant anagen in E2F4$^{-/-}$(n=12) and Rb$^{f/f}$;K14creER^{TM};E2F4$^{-/-}$mice (n=10). Paired littermates were analyzed at 4 and 12 months after tamoxifen treatment. Bars= 50 µm (B, B' Bars= 200 µm).

Supplementary Table S1: Genes deregulated between Rb$^{f/f}$;K14creER^{TM};E2F4$^{-/-}$ and Rb$^{f/f}$;K14creER^{TM} skin.

See Supplementary File 1

Supplementary Table S2: Genes deregulated between Rb$^{f/f}$;K14creER^{TM};E2F4$^{-/-}$ and Rb$^{f/f}$;K14creER^{TM};E2F1$^{-/-}$ skin.

See Supplementary File 2
